# What's new in emergencies, trauma and shock? Publishing clinical trials in JETS from countries around the globe

**DOI:** 10.4103/0974-2700.44673

**Published:** 2009

**Authors:** Veronica Tucci, Sagar Galwankar, Tracy Sanson, Kelly O'Keefe

**Affiliations:** University of South Florida, College of Medicine, Tampa, Florida USA

The art of medicine cannot advance without the tireless efforts of physician-scientists and the commitment and generosity of our patients who agree to participate in clinical trials. Unfortunately, not all original research conforms to the highest ethical standards and patients frequently suffer despite all good intentions. Institutional Review Boards, Hospital and Professional Ethics Committees have attempted to curb abuse and maintain the public trust. However, these bodies alone while essential to promoting quality scientific research cannot ensure transparency and honesty. Recent reports have questioned the objectivity of the research and highlighted unethical conduct in reporting of clinical results or the omission thereof. To this end, journals around the globe are now requiring clinical trials to be registered in a public registry. JETS now joins our sister journals in requiring that physician-scientists prospectively register clinical trials to promote transparent clinical research and scientific integrity.

Since virtually the dawn of recorded time, scientists and physicians have utilized clinical trials to improve patient care and advance the art of medicine. From Dr. Mengele to the Tuskegee Institute, history has been rife with examples of unethical, scientifically questionable, and even immoral experiments with the concomitant abominable treatment of patients enrolled in these “clinical trials”. Unfortunately, even with the adoption of the Nuremberg Codes, the Declaration of Helsinki and other standards for the ethical conduct and informed consent, new problems have surfaced in the arena of evidence-based medicine and the collection of data through clinical trials.

Recent reports have questioned whether clinical trials are failing to advance the science of medicine by objectively and honestly reporting the results of the experiments.[1] Instances of selective reporting or omission based upon the outcome of the trial and/or when conflicting financial interests are involved color the scientific process and hinder our ability to investigate new treatments, methods/protocols and undermine the trust that the public places in our profession.[2]

Despite our best efforts to ensure transparency and honesty, it is becoming increasingly clear that initiatives to discourage unethical conduct in the reporting of or the failure to report study results have largely been unsuccessful.[3]

Moreover pharmaceutical companies and other agencies who are less than eager to report unfavorable data have resisted efforts to establish public registries for clinical trials.[4] This reluctance is further complicated by the fact that trial registration is voluntary.[5]

To protect both our patients and the integrity of the scientific process, JETS joins the International Committee of Medical Journal Editors (ICMJE), the World Association of Medical Editors and our sister journals including the Journal of the American Medical Association,[6] Annals of Internal Medicine, British Medical Journal, Canadian Medical Association Journal, Croatian Medical Journal, Journal of the American Medical Association, The Dutch Medical Journal (Nederlands Tijdschrift voor Geneeskunde), New England Journal of Medicine, New Zealand Medical Journal, The Lancet, The Medical Journal of Australia, Tidsskrift for Den Norske Laegeforening, Journal of the Danish Medical Association in calling for the registration of all clinical trials in a public registry (as set forth in the guidelines below) as an absolute condition before a manuscript will be considered for publication.[7]

JETS defines a clinical trial as any study that prospectively assigns human subjects to either an intervention or comparison group to evaluate the causal relationship between a medical intervention and a health or biomedical outcome/end-point.[7,8]

JETS defines interventions as any medical, surgical, psychological or sociological procedure, therapy or treatment including but not limited to medications, herbal, natural or home remedies, use of therapies including acupuncture, coining, cupping, and other traditional Eastern remedies, orthopedic or chiropractic manipulation as well as the use of imaging studies such as X-rays, ultrasound, or any other modality designed or utilized by medical personnel for clinical assessment, decision-making and treatment/disposition.[7,8]

Several interventions may be compared simultaneously, or one or more treatment groups may be compared to a simultaneous, untreated control group. Although the division into such groups in most such trials is presently by random assignment, randomization is not a requirement for registration.

Studies designed for other purposes including Phase I trials that examine pharmacokinetics or investigate major drug toxicities are exempt from the registration requirements set forth herein. Similarly, retrospective analyses and the mention of clinical trials in any case report, case series, literature reviews will be exempt from the registration requirement.

The size of a clinical trial is irrelevant for purposes of ascertaining whether it must be registered prior to manuscript submission. This policy applies not only to large, multi-institutional clinical trials sponsored by pharmaceutical companies or other organizations, but also to individual investigators at a single institution who are conducting their own trials.

## GUIDELINES ON CLINICAL TRIALS REGISTRATION FOR THE JOURNAL OF EMERGENCIES, TRAUMA AND SHOCK (JETS)

1. Beginning January 1, 2010, JETS will not solicit, review, accept or publish any papers dealing with clinical trials that were not registered prospectively with a trial registry prior to the enrollment of the study's first subject.
2. From the date of publication of this clinical trials policy through December 31, 2009, all trials that are not prospectively registered must be registered retrospectively with a trial registry before they will be considered for publication.
3. A letter accompanying all manuscript submissions regarding clinical trials must state the beginning date of the trial, specify the trial registry, the assigned number and the date the trial was registered with that specific registry.
4. Although a universal, worldwide registry for clinical trials would be ideal, at present, JETS will accept registration of a clinical trial with one of the following publicly available registries:
  - NIH's registry http://www.clinicaltrials.gov
  - World Health organization, International Clinical Trials Registry Platform (ICTRP). http://www.who.int/ictrp/en/
  - The International Standard Randomized Controlled Trials Number (http://isrctn.org).
  - Australian Clinical Trials Registry http://actr.org.au
  - Nederlands Trial Register http://www.trialregister.nl/trialreg/index.asp
  - UMIN Clinical Trials Registry http://www.umin.ac.jp/ctr
  - The Clinical Trial Registry India www.ctri.in
  - http://www.chictr.org/ (China)
  - The list of approved registration sites will be updated periodically at onlinejets.org

It was unanimously decided that the editors have the responsibility to promote the registration of all clinical trials in the field of emergency medicine being conducted in the United States and abroad and to urge researchers and physician-scientists to register their trials within the stipulated time frame above, to make the clinical trial data transparent and to enable results to be published in good journals.

JETS urges physician investigators and all other personnel involved in planning, conducting or otherwise involved in clinical trials involving human subjects, to register their studies with a clinical trial registry and to comport their clinical research activities with the highest degree of integrity, keeping with the tenets of our profession and maintaining the public's trust.

Any inquiries regarding this policy or future updates should be directed to editor@onlinejets.org.
